# Solidified Reverse Micellar Solution- (SRMS-) Based Microparticles for Enhanced Oral Bioavailability and Systemic Antifungal Efficacy of Miconazole Nitrate in Immunocompromised Mice

**DOI:** 10.1155/2022/8930709

**Published:** 2022-01-25

**Authors:** Emmanuel Maduabuchi Uronnachi, Anthony Attama, Franklin Kenechukwu, Chukwuebuka Umeyor, Thaddeus Gugu, Calistus Nwakile, Chidalu Ikeotuonye

**Affiliations:** ^1^Nanomedicines and Drug Delivery Research Group, Department of Pharmaceutics and Pharmaceutical Technology, Nnamdi Azikiwe University, Awka, 422001 Anambra State, Nigeria; ^2^Drug Delivery and Nanomedicines Research Group, Department of Pharmaceutics, University of Nigeria, Nsukka, 410001 Enugu State, Nigeria; ^3^Department of Pharmaceutical Microbiology and Biotechnology, University of Nigeria, Nsukka, 410001 Enugu State, Nigeria

## Abstract

**Purpose:**

To assess the improvement in oral bioavailability and efficacy in systemic candidiasis treatment of miconazole nitrate (MN) formulations in murine models of candidiasis.

**Methods:**

Selected formulations containing 5% of Softisan + Phospholipon 90H lipid matrix with 3% of MN (*A*_1_), 5% of stearic acid + Phospholipon 90H lipid matrix with 3% of MN (*B*_1_), and 5% Softisan + stearic acid + Phospholipon 90H with 3% of MN (*C*_1_) from the *in vitro* investigation were used for the study. Their acute toxicity was assessed using Lorke's method (with slight modification) while bioavailability was determined using the bioassay method. The optimized batch (*A*_1_) was tested in murine systemic candidiasis induced in cyclophosphamide-immunosuppressed mice. The mice were treated with a single oral dose (100 mg/kg) of the formulations for five days. Serum fungal counts (cfu/mL) were determined on days 1, 3, and 5 of the treatment period. Haematological assessments were done.

**Results:**

The lipid formulations were safer than MN powder with LD_50_ values of 3162.8 and 1118.3 mg/kg. Bioavailability determination revealed a higher area under the curve (AUC) value for formulations *A*_1_ (6.11 *μ*g/hr/mL) and *B*_1_ (4.91 *μ*g/hr/mL) while formulation *C*_1_ (1.80 *μ*g/hr/mL) had a lower AUC than MN (4.46 *μ*g/hr/mL). Fungi were completely cleared from the blood of animals treated with the optimized formulation by day 3 as opposed to the controls (MN and Tween® 20) which still had fungi on day 5. No significant increase (*p* > 0.05) in haematological parameters was observed in mice treated with *A*_1_.

**Conclusion:**

Formulation *A*_1_ successfully cleared *Candida albicans* from the blood within a shorter period than miconazole powder. This research has shown the potential of orally administered MN-loaded SRMS-based microparticles in combating systemic candidaemia.

## 1. Introduction

Candida species are harmless commensal organisms that are regularly found in the normal flora of humans. However, in immunocompromised and immunologically weak persons, they become opportunistic pathogens causing harm to their host [1]. Common diseases caused by Candida species include vulvovaginal candidiasis, oropharyngeal candidiasis, and systemic candidiasis or disseminated candidaemia. The most common causative organism of systemic candidiasis among the Candida spp. is *Candida albicans* [1]. Candida infection is generally classified as superficial (affecting the skin, nails, oropharynx, vagina, oesophagus, and gastrointestinal tract) or deep (affecting the blood and other organs of the body).

The management of systemic candidiasis has posed some challenge to therapists due to the limited number of drug options available. Some of the desirable features of potential antifungal agents for treating systemic candidiasis include a broad spectrum of activity, effective tissue penetration, a good safety profile, low potential for toxicity, and ease of administration [2]. Among the therapeutic agents available, cost and toxicity are their obvious limitations. These limitations have prompted several researchers [3–5] to explore formulations that would lower the burden of cost and toxicity as well as enhance the arsenal of drug options available to combat the disease.

Lipids have increasingly become candidates for drug delivery due to their biocompatibility and low toxicity. The size of the system affects its functionality with lipid microparticles having the advantages of ease of production and characterization, possibility of an extended release profile, and a relatively high drug loading which causes a reduction in quantity administered [6]. Furthermore, solid lipid microparticles are more advantageous than solid lipid nanoparticles in drug delivery due to a prolonged drug release leading to an increased bioavailability [7]. In addition, they possess particle sizes larger than 100 nm which prevents uptake by the reticuloendothelial system (RES), can encapsulate toxic substances as well as liquids (in solid form) as dried microparticles, and can be utilized in multifunctional drug delivery where small particles are encapsulated as separate units thus ensuring release at predetermined times and manner [8]. Several mechanisms have been suggested for the ability of lipids to enhance bioavailability. These include increasing membrane fluidity thereby facilitating transcellular absorption, stimulation of lipoprotein/chylomicron production, enhancing lymphatic uptake, and systemic delivery of drugs via the lymphatic route [9].

Several authors have explored the potential use and applicability of lipid microparticles in drug delivery for enhancing the bioavailability of poorly soluble therapeutic molecules. Kenechukwu et al. prepared solid lipid microparticles containing gentamicin (a poorly absorbed drug) using Phospholipon® 90G and dika wax. Their formulation increased oral bioavailability of gentamicin 2.2-fold [10]. Also, Hussain et al. have presented several microparticle-based lipid formulations that enhanced the oral bioavailability of poorly soluble drugs [11].

Miconazole is an azole antifungal belonging to the imidazole class but has a poor aqueous solubility (<1 *μ*g/mL) thus making it poorly bioavailable [12]. It acts via two mechanisms: inhibition of ergosterol biosynthesis and inhibition of lipid peroxidases that cause an accumulation of peroxides in the cell thereby causing cell death [13, 14]. This limitation of poor aqueous solubility precludes its oral use in the treatment of systemic fungal diseases like systemic candidiasis and candidaemia. It is however potent in the treatment of other forms of fungal diseases, e.g., vaginal candidiasis, superficial candidiasis, dermatophytosis, and pityriasis versicolor [15]. Its potency and long half-life make it a promising candidate for the treatment of systemic fungal diseases.

In the intravenous delivery of miconazole, castor oil has been used as a delivery vehicle to enhance solubilization and delivery. However, this formulation was associated with toxicities linked to the vehicle-castor oil. In addition, due to the short duration of therapy occasioned by this route of administration, there have been incidences of high relapse rates when employed [2]. Oral drug administration has several advantages over other routes: patient friendliness, convenience, cost effectiveness, and noninvasiveness [16]. Consequently, several authors have worked on formulations to improve oral bioavailability of miconazole nitrate with mixed results [12, 17].

Previous studies have established an improvement in the *in vitro* release of miconazole nitrate from solidified reverse micellar microparticles [18]. Nonetheless, in order to further improve the oral administration as well as establish the usefulness of the microparticles *in vivo*, the present work evaluated the pharmacodynamic and pharmacokinetic properties of these microparticles containing miconazole nitrate in treating systemic candidaemia. The novelty embodied in the study is the use (for the first time) of solidified reverse micellar solution- (SRMS-) based microparticles to enhance the systemic circulation longevity of miconazole while ameliorating its systemic adverse effects. Hypothetically, the use of SRMS-based microparticles would enhance the oral bioavailability as well as the therapeutic potential of miconazole nitrate in the treatment of systemic candidiasis, with added merits of less potential for resistance and recurrence.

Consequently, the objectives of this study were to optimize SRMS-based microparticles containing miconazole nitrate and evaluate the optimized formulations for enhanced oral bioavailability in the treatment of systemic candidiasis in a murine model.

## 2. Materials

The following materials were used as procured from their manufacturers without further purification: miconazole nitrate (Gutic Biosciences, India), stearic acid (Spectrum Lab, India), Softisan® 154 (Cremer Oleo, GmbH, Germany), Tween® 20 (Merck, Darmstadt, Germany), sorbic acid (Qualikems, India), methanol (Sigma-Aldrich, England), Phospholipon® 90H (Phospholipid GmbH, Germany), and distilled water (Pauco Pharmaceuticals, Awka, Nigeria). Other reagents and materials used were of analytical grade.

## 3. Methodology

### 3.1. Preliminary Formulation of Lipid Matrices

Lipid matrices (LM) were formulated by weighing out specific quantities (Table 1) of the lipid and Phospholipon® 90H and heating to a temperature above the melting points of the respective lipids to ensure melting in a water bath.

When they were sufficiently melted, they were stirred continuously until they thickened. The formed solid lipid matrices were then transferred to different containers and stored.

### 3.2. Formulation of Microparticles Using Optimized Parameters

A surfactant concentration of 1.5%, homogenization speed of 5000 rpm, and homogenization time of 5 min were experimentally determined to be the optimum parameters for the study. In the preformulation studies, different surfactant concentrations (1%, 1.5%, and 2%), different homogenization speeds (5000, 10,000, 15,000, and 20,000 rpm), and different time intervals (5, 10, 15, and 20 min) were studied. Among these combinations, the combination that gave a stable preparation (absence of creaming, colour change, congealing, and separation) after one month of evaluation was chosen for the study. The melt homogenization method was used for the formulation. Briefly, 5 g of lipid matrix was weighed out and heated on the temperature controlled magnetic stirrer (Ika, Germany). The required quantities (Table 2) of the other excipients (sorbitol, sorbic acid, and Tween® 80) were weighed out, dispersed in about 70 mL of water, and also placed on the magnetic stirrer and allowed to heat together with the lipid matrix until it melted. After melting, 3 g of miconazole nitrate was weighed out and dispersed in the melted lipid matrix. The aqueous portion was then added to the lipid portion still on the stirrer and homogenized at 5000 rpm for 5 min.

After preparing the formulations, they were allowed to stand at room temperature for 24 h to allow for complete recrystallization of the microparticles, after which they were freeze dried using a Christ Beta 1-8 LD plus (UK) freeze drier at a temperature range of -50 to -60°C and a pressure of 100–300 torr. Sorbitol was used as the cryoprotectant.

The freeze-dried solid lipid microparticles were kept in the refrigerator at 4°C.

### 3.3. Determination of Calibration Curve and Minimum Inhibitory Concentration (MIC) of Miconazole for *Candida albicans*

Different concentrations of miconazole ranging from 0.125 *μ*g/mL to 16 *μ*g/mL (twofold increases) were prepared using 1% *v*/*v* Tween® 20. A 100 *μ*L volume of the prepared concentrations was then placed in holes bored on agar plates streaked with *Candida albicans*, allowed for a period of 30 min for prediffusion, and then incubated at 25°C for 48 h. At the end of the period, the inhibition zone diameter (IZD) of the various concentrations was measured. The solvent for dilution (1% Tween® 20 solution) served as the blank for the experiment.

### 3.4. Acute Toxicity Determination of Freeze-Dried Microparticles

Representative batches from the formulations were chosen for the study. All animal experiments were carried out in accordance with the guidelines of the Animal Ethics Committee of the Faculty of Pharmaceutical Sciences, Nnamdi Azikiwe University, Awka, Nigeria, and EU directive 2010/63/EU for animal experiments.

This test was carried out using Lorke's method [19] with modifications. Forty mice of both sexes weighing 17-30 g were used for this study. They were placed in cages and allowed access to food and water for a period of one week for acclimatization, then fasted overnight before being administered with the formulations equivalent to the following doses of the drug: 100, 300, 1000, 2000, and 5000 mg/kg.

These were dosed with representative drug samples for the formulations containing the different lipid combinations. Those chosen contained the highest entrapped drugs for their respective lipid combinations: 5% LM_1_ containing 3% of MN (*A*_1_), 5% LM_2_ containing 3% of MN (*B*_1_), and 5% LM_3_ containing 3% of MN (*C*_1_).

For each of the three dose groups, three mice were used for the 100, 300, and 1000 mg doses, respectively. One animal alone was used in each group for the 2000 and 5000 mg/kg dose.

After administration, the animals were allowed free access to food and water again and observed for a 24 h period. At the end of the period, the number of deaths was recorded for each dose category.

### 3.5. Bioavailability Studies of Formulation in Rats

A concentration of 100 mg/kg of the formulation was experimentally determined to be suitable for this experiment using the inhibition zone diameter (IZD) method for determining serum drug concentration.

For this study, the three batches of the formulations (*A*_1_, *B*_1_, and *C*_1_) were used plus miconazole nitrate (MN) powder as the control. Twenty-four (24) Wister rats weighing 100–150 g were used for the study. They were placed in groups of six and allowed free access to food and water as well as observing adequate light and dark cycles for a period of one week to acclimatize. After the acclimatization process, a dose of the formulation containing 100 mg/kg of the miconazole nitrate was administered to the animals in each group. Blood (1 mL volumes) was then withdrawn from the retroorbital plexus of the rats using heparinized capillary tubes at intervals of 0, 0.5, 1, 2, 4, and 8 h, respectively. The withdrawn blood was centrifuged using a refrigerated centrifuge (TGL-20M, China) at a speed of 400 × *g* for 10 min. After centrifugation, the serum was collected using a micropipette, placed in Eppendorf tubes, and refrigerated at 4°C until needed.

### 3.6. IZD Determination of Drug in Serum

Drug concentration in the serum collected at different time points was determined by bioassay. Sterile Petri dishes were collected, and molten sabouraud dextrose agar was poured into them. They were then inoculated with *Candida albicans* strains (0.5 McFarland's standard) and allowed to set. Afterwards, a sterile cork borer with a diameter of 8 mm was used to bore holes on the agar. A 100 *μ*L volume of the serum was aseptically placed in the bored holes and allowed for a period of 30 min for prediffusion, before incubating at 25°C for 48 h. At the end of the period, the developed IZDs were measured. These values were fitted to the calibration curve obtained for pure miconazole nitrate against *Candida albicans* to determine their effective serum concentrations.

### 3.7. Determination of the Pharmacokinetic Parameters of Formulations Administered *In Vivo*

The pharmacokinetic parameters: maximum serum concentration (*C*_max_), time to reach maximum serum concentration (*T*_max_), area under the plasma concentration time curve (AUC), area under the first moment curve (AUMC), plasma half-life (*T*_1/2_), volume of distribution (*V*_*d*_), clearance, and mean residence time (MRT) of the formulations administered *in vivo* were determined using the software WinNonLin 5.0 (Pharsight Corp., USA).

### 3.8. Assessment of Drug Effect in Immune-Compromised Mice Infected with *Candida albicans*

This was done in several stages.

#### 3.8.1. Stage 1: Induction and Assessment of Immune Suppression in Experimental Animals

Here, the method of Hussain et al. [20] with slight modification was used. Two groups (A and B) of five (5) mice each of both sexes were used. They were both administered a single dose of cyclophosphamide (50 mg/kg) intraperitoneally and left for 72 h with free access to food and water. White blood cell counts of the mice were taken before and after 72 h of drug administration to confirm immune suppression.

#### 3.8.2. Stage 2: Induction of Candidiasis in Immune-Compromised and Immune-Competent Animals

Three groups of mice (each containing five animals) were used for this study. The organism (*Candida albicans*) was administered via the tail vein. Groups 1 and 2 were the immune-compromised animals previously administered a single dose of cyclophosphamide. Group 3 animals were immune-competent.

#### 3.8.3. Stage 3: Antifungal Therapy

The three groups of animals induced with *Candida albicans* were used. Drug therapy commenced 24 h after induction with *Candida albicans*. Group 1 received a daily dose of 100 mg/kg of miconazole nitrate powder solubilized with 1% Tween® 20 for five days. Group 2 received a daily dose of 100 mg/kg of the optimized formulation (*A*_1_) for five days. Group 3 received 0.5 mL of a 1% *v*/*v* Tween® 20 solution used to solubilize the pure drug and served as the negative control. Blood samples were collected at days 1, 3, and 5 from animals in each group and assayed microbiologically for quantitative enumeration of colony-forming units (cfu) of *Candida albicans*.

### 3.9. Haematological Studies

Blood samples were collected from the animals prior to commencement of therapy, i.e., on day 0 and after completion of therapy, i.e., on day 6. These samples were analyzed using a Sysmet 3-part differential automated analyzer (Sysmet, USA) for the following parameters: white blood cell (WBC) count, haemoglobin, packed cell volume (PCV), mean corpuscular volume (MCV), mean corpuscular haemoglobin (MCH), monocytes, platelets, lymphocytes, and neutrophils.

### 3.10. Statistical Analysis

The results obtained were presented as the mean ± standard deviation (SD) using Microsoft Excel 2013 and WinNonLin 5.0 (Pharsight Corp., USA). The data were subjected to one-way analysis of variance (ANOVA), and group differences were determined using post hoc least significant difference (LSD) multiple comparisons' test using SPSS version 16. Results were considered statistically significant at *p* < 0.05.

## 4. Results and Discussion

### 4.1. Lipid Matrix Formulation

The formation of solidified reverse micelles is achieved using a combination of phospholipids (30-60%) and triglycerides or hard fats as shown in studies by Friedrich and Müller-Goymann [21]. Also, previous studies have demonstrated the effectiveness of this phospholipid concentration in forming solidified reverse micellar solution- (SRMS-) based microparticles [10]. While the study by Friedrich and Müller-Goymann illustrated an increased drug loading with higher phospholipid concentrations (e.g., 50%), it has also been demonstrated that this difference may not be hugely significant, e.g., in the work of Chime et al. [22]. Hence, we resorted to using a constant concentration of 30% for the phospholipid in the lipid matrix.

### 4.2. Acute Toxicity Evaluation

From the acute toxicity results obtained (Table 3), it was observed that doses of up to 2000 mg/kg of all the formulations were safe since no death was recorded, while at a dose of 5000 mg/kg, all the animals died. For the pure miconazole nitrate drug, doses as low as 1000 mg/kg caused death in the animals thus indicating a greater toxic effect. The difference in toxicities of the formulations and the pure drug may have arisen from the presence of a lipid core surrounding the drug particles in the microparticle formulation. This core may have delayed drug release thus limiting the effects of dose dumping and its associated toxicities. Some authors have reported that the surface area of SLMs and their diffusion length from the core of the lipid matrix to the surface of the particle may affect the release rate of drugs from particles [23].

### 4.3. Bioavailability Determination of Selected Microparticles

**Figure 1:**
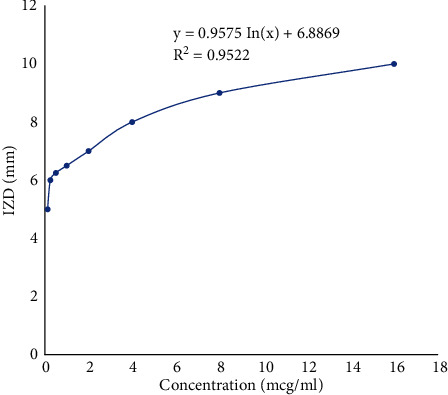
Calibration plot of miconazole nitrate against *Candida albicans*.

**Figure 2:**
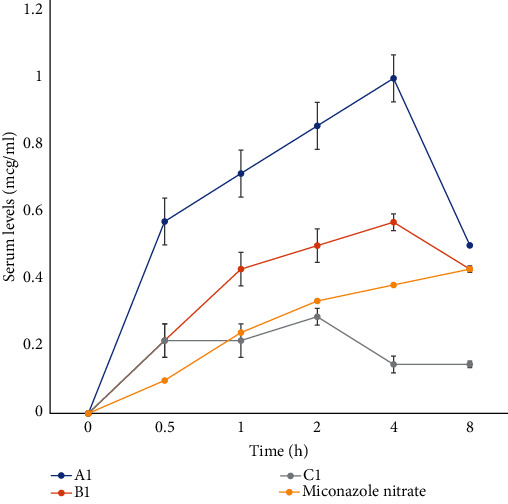
Bioavailability determination of selected formulations.

The calibration plot used to determine the serum concentrations of miconazole nitrate is represented in Figure 1. The bioassay method of drug quantification in the plasma is a simple and efficient method of analysis. It has been reported by several researchers as a reliable and comparative tool to the High-Performance Liquid Chromatography (HPLC) method in quantifying plasma drug content especially for antimicrobial agents. Zuluaga et al. [24] demonstrated the efficacy of using the microbiological assay method to quantify antibiotics in the plasma. Their method validation showed a high linearity, precision, accuracy, and specificity for the microbiological assay method. Manfio et al. [25] also evaluated the potency of ceftriaxone sodium using the microbiological assay method. Their results were comparable to that obtained using liquid chromatography method. Cendejas-Bueno et al. [26] demonstrated the validity of the bioassay method as an alternative to HPLC/UV analysis in the quantification of posaconazole in the human serum. Umeyor et al. [27] successfully quantified gentamicin in the serum using the bioassay method. The results of the bioavailability determination are represented graphically as shown in Figure 2. The time-dependent serum concentration values when fitted into pharmacokinetic software (WinNonLin version 5.0, Pharsight Corp., USA) yielded the data in Table 4.

A statistical comparison of the pharmacokinetic parameters of the different formulation batches with that of miconazole powder showed that *C*_max_ was significantly different (*p* < 0.05) for Batch *A*_1_ while the other two batches were not significantly different (*p* > 0.05) from miconazole. In addition, the AUC, *T*_1/2_, clearance, MRT, and AUMC were all significantly different from the results obtained for miconazole for all the batches, while the *V*_*d*_ of Batches *A*_1_ and *B*_1_ alone were significantly (*p* < 0.05) different from that of miconazole powder.

The formulation with the least *C*_max_ was Batch *C*_1_ with a value of 0.29 *μ*g/mL while Batch *A*_1_ had the highest *C*_max_ of 1.0 *μ*g/mL. This was much higher than the *C*_max_ of MN powder with a value of 0.43 *μ*g/mL. Lipids have an inherent ability of increasing the GIT absorption of drugs by the formation of micelles which promote solubilization and subsequent absorption.

The time to reach maximum concentration (*T*_max_) of miconazole powder was 8 h while the other formulations had a lower *T*_max_ of 2 h for Batch *C*_1_ and 4 h for Batches *A*_1_ and *B*_1_. This could be due to a greater bioavailability of the drug in the system caused by an improved absorption because of the lipids used in formulating the drug.

AUC is dependent on systemic drug concentration and drug clearance from the systemic circulation [28]. A cursory look at the AUC and clearance values for the formulations and the pure MN indicated lower clearance values for formulations with large AUC values and vice versa. For instance, Batch *A*_1_ had the highest AUC value of 6.11 *μ*g h/mL and had the least clearance value of 0.70 mL/kg/h. Batch *C*_1_ had the least AUC value of 1.8 *μ*g h/mL with the greatest clearance value of 5.04 mL/kg/h. MN, on the other hand, had an AUC of 4.46 *μ*g h/mL and a clearance of 0.88 mL/kg/h.

The low clearance of MN may have been occasioned by its poor water solubility which can lower its kidney excretion and high lipophilicity as seen in its high octanol-water partition coefficient of 6.25 [29]. This reduced excretion is evidenced in its large terminal half-life of 20.52 h, which was significantly higher than that of the other formulations. There was an obvious correlation between the terminal half-lives of the formulations and their clearance values with higher half-lives corresponding to lower clearance values and vice versa.

Clearance of a drug is useful in determining the maintenance dose required to obtain a steady state serum concentration of the drug [30] as well as in the evaluation of drug elimination from the kidney. The clearance represents the theoretical volume of blood or plasma, which is cleared of the drug in a given period [31]. It can also be defined as the volume of blood cleared completely of drug per unit time (L/h or mL/min).

Also, the mean residence time (MRT) of MN powder was the highest with a value of 6.70 h while the Batch *A*_1_ formulation had the least MRT of 3.81 h, showing a possible distribution of the drug from the plasma to the tissues (Table 4). This property becomes increasingly important in tackling systemic candidiasis since the organism is cleared rapidly from the plasma and concentrates in the tissues and organs, e.g., the liver, spleen, and kidney.

The volume of distribution values was typically low for all the formulations. This may be because miconazole is highly protein bound (about 99%). High protein binding often leads to a lower volume of distribution. Consequently, the MN powder had a volume of distribution of 26.02 mL; Batch *C*_1_ had a *V*_*d*_ of 27.24 mL; Batch *B*_1_ had a *V*_*d*_ of 10.31 mL, while Batch *A*_1_ had a *V*_*d*_ of 11.47 mL (Table 4). The volume of distribution is the hypothetical volume within which a drug is distributed in the body [28]. This volume can be very small if the drug is primarily contained in the blood or very large if the drug is redistributed widely in the body and is mostly bound to body tissues [32].

### 4.4. WBC Counts of Mice during Induction of Immune Suppression

The results obtained (Table 5) indicated that there was neutropenia after the third day of induction. Neutropenia (low white blood cell count) is a confirmation of immune suppression [5].

### 4.5. Assessment of Drug Effect on Candidaemia

**Figure 3:**
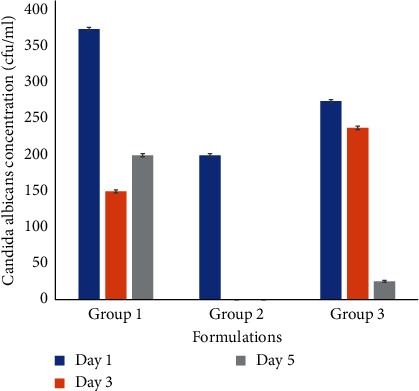
Blood colony counts of *Candida albicans* in immune suppressed mice. Group 1: immunosuppressed mice induced with candidiasis and administered miconazole solution (100 mg/kg). Group 2: immunosuppressed mice induced with candidiasis and administered Batch *A*_1_ (100 mg/kg). Group 3: mice induced with candidiasis and administered 1% Tween® 20 solution.

The results of Figure 3 showed a low fungal count on day 1 for the animals administered the optimized formulation (*A*_1_) while fungal counts for miconazole and Tween® 20 were higher. By day 3, however, there were no fungi in the blood of the animals administered the optimized formulation as compared with the animals administered miconazole nitrate alone or with the vehicle (Tween® 20). This clearance of fungi from the blood could be because of a higher drug concentration of miconazole nitrate from the formulation as evidenced in the bioavailability studies. Furthermore, for a drug to effectively clear microorganisms in the blood, its blood concentration must be higher than the MIC of the drug for the organisms. The bioavailability results obtained showed a *C*_max_ of 1 *μ*g/mL for the Batch *A*_1_. This concentration is higher than the MIC of miconazole which has been reported by several researchers to range from 0.12 *μ*g/mL (for sensitive organisms) to 0.5 *μ*g/mL (for fluconazole resistant C. albicans) [33] and 0.063-0.125 *μ*g/mL [34]. Because of a higher plasma level, the clearance of the organism from the blood was accomplished by day 3 postadministration. Other agents administered showed reductions in blood counts of the organism. This may be due to the presence of suboptimal concentrations of the drug in the blood (for group 1 animals administered the miconazole nitrate solution) or the body's innate immune system for group 3 animals administered the vehicle (1% Tween® 20 solution). The result further highlights the advantage of the formulation over conventional oral administration of the drug.

### 4.6. Haematological Effect

From Table 6, the analysis of variance (ANOVA) at a level of significance of *p* < 0.05 revealed the following: there was a significant difference in the PCV values between groups (*p* ≤ 0.001), the TWBC count between groups was also significant (*p* = 0.040), MCHC between groups was significant (*p* = 0.025), MCH between groups was insignificant (*p* = 0.459), MCV between groups was significant (*p* = 0.040), PLT between groups was significant (*p* ≤ 0.001), Hb between groups was also significant (*p* = 0.002), lymphocyte between groups was significant (*p* ≤ 0.001), neutrophil between groups was significant too (*p* ≤ 0.001), and monocyte between groups was insignificant (*p* = 0.393).

Further analysis using the least significance difference (LSD) post hoc test revealed the following: for the PCV results, a significant difference existed in group 1 between pre and post values (*p* ≤ 0.001), while the other groups showed no significant differences between their “pre” and “post” values, for group 2 (*p* = 0.857) and group 3 (*p* = 0.119).

For the MCHC results, the pre and post results were significant for groups 1 (*p* = 0.004) and 3 (*p* = 0.015), while those of group 2 were insignificant (*p* = 0.255).

For the Hb values, there was a significant difference in the pre and post values for group 1 (*p* = 0.002). The remaining groups had no significant difference in their values (*p* > 0.05) and were as follows: group 2 (*p* = 0.961) and group 3 (*p* = 0.084).

For the lymphocyte count, there was significant difference between the pre and post values of group 1 (*p* ≤ 0.001), while the remaining groups had insignificant differences between their pre and post values, with values of *p* = 0.353 and *p* = 0.930 for groups 2 and 3, respectively.

For the neutrophil count, the only significant values were those of group 1 (*p* ≤ 0.001). For other groups, their values were as follows: *p* = 0.648 and *p* = 0.701 for groups 2 and 3, respectively.

The body has a defense mechanism against diseases and infections. This is often seen in the release of white blood cells and inflammatory mediators to the site of the infection to fight such diseases. In some disease states, prevalence of certain types of white blood cells could be indicative of the presence of such an infection. From the results obtained, there were significant elevations in PCV, MCHC, Hb, lymphocyte, and neutrophil counts in the group administered with the miconazole nitrate solution alone in comparison with the group administered with the optimized formulation. These increases may be because of the combined effect of the disease state in the animals and drug concentration, which may not have effectively inhibited the disease. Neutrophils are usually the first to be released to the site of an infection [35]. Martinez-Rossi et al. [36] reported a high neutrophil count in diseased tissues containing *C. albicans*. In addition, Tuzcu et al. [37] reported a high neutrophil count in mice infected with fungi while Mohammed et al. [38] reported an increase in haematological parameters in patients and rabbits with Candida infection.

On the other hand, the group administered the optimized formulation and did not record any significant alterations in their haematological parameters before and after drug administration. This suggests a good safety profile of the formulation and further supports the results obtained from the acute toxicity evaluation.

## 5. Conclusion

Miconazole nitrate-loaded SRMS microparticles were successfully formulated and evaluated *in vivo* for treatment of candidaemia. The optimized formulation (*A*_1_) showed better pharmacokinetic properties and successfully cleared *Candida albicans* from the blood within three days in comparison with the pure drug (unformulated) that was unable to do so even after a five-day period. The research showed the promising nature of the oral delivery of miconazole nitrate using SRMS-based microparticles for combating candidaemia.

## Figures and Tables

**Table 1:** Formulation composition of the lipid matrices.

Formulation	Percentage ratio		
	Phospholipon® 90H	Softisan® 154	Stearic acid
LM_1_	30% *w*/*w*	70% *w*/*w*	—
LM_2_	30% *w*/*w*	—	70% *w*/*w*
LM_3_	30% *w*/*w*	35% *w*/*w*	35% *w*/*w*

LM: lipid matrix.

**Table 2:** Composition of solid lipid microparticles (SLMs).

Content	Percentage composition (%)
Lipid matrix	5.0
Polysorbate 80	1.5
Drug	3
Sorbic acid	0.2
Sorbitol	4.0
Water	100

**Table 3:** Acute toxicity determination for selected microparticle batches.

Formulation	Highest safe dose (mg/kg)	Lethal dose (mg/kg)	LD_50_ (mg/kg)
Batch *A*_1_	2000	5000	3162.68
Batch *B*_1_	2000	5000	3162.68
Batch *C*_1_	2000	5000	3162.68
Miconazole nitrate	500	1000	1118.03

MN: miconazole nitrate; Batch *A*_1_: 5% LM_1_, containing 3% MN; Batch *B*_1_: 5% LM_2_ containing 3% MN; Batch *C*_1_: 5% LM_3_ containing 3% MN.

**Table 4:** Pharmacokinetic parameters of formulations.

Formulation	*C*_max_ (*μ*g/mL)	*T*_max_ (hr)	AUC (*μ*g/hr/mL)	AUMC (*μ*g/mL/hr^2^)	*T*_1/2_ (hr)	*V*_*d*_ (mL)	Clearance (mL/kg/hr)	MRT (hr)
Batch *A*_1_	1.00 ± 0.13	4.00 ± 0.18	6.11 ± 0.11	23.25 ± 1.78	11.42 ± 1.2	11.47 ± 0.23	0.70 ± 0.09	3.81 ± 0.14
Batch *B*_1_	0.57 ± 0.06	4.00 ± 0.15	4.91 ± 0.13	26.08 ± 3.22	4.15 ± 0.21	10.31 ± 0.23	1.72 ± 0.17	5.31 ± 0.67
Batch *C*_1_	0.29 ± 0.05	2.00 ± 0.34	1.80 ± 0.09	8.35 ± 0.89	3.75 ± 0.14	27.24 ± 3.45	5.04 ± 0.88	4.65 ± 0.11
MN powder	0.43 ± 0.08	8.00 ± 0.74	4.46 ± 0.06	29.89 ± 2.45	20.52 ± 1.37	26.02 ± 2.89	0.88 ± 0.09	6.70 ± 0.45

MN: miconazole nitrate; Batch *A*_1_: 5% LM_1_, containing 3% MN; Batch *B*_1_: 5% LM_2_ containing 3% MN; Batch *C*_1_: 5% LM_3_ containing 3% MN (values presented as the mean ± SD, *n* = 3).

**Table 5:** Results of WBC count of mice injected with cyclophosphamide solution (50 mg/kg).

Group	WBC count preinduction × 10^3^ (mm^3^)	WBC count post induction × 10^3^ (mm^3^)
Group 1	7.60 ± 1.84	2.00 ± 0.63
Group 2	8.50 ± 1.51	2.00 ± 1.00

Values presented as the mean ± SD (*n* = 3).

**Table 6:** Results of haematological parameters of infected mice before and after treatment.

Parameter	Group 1 (pre)	Group 1 (post)	Group 2 (pre)	Group 2 (post)	Group 3 (pre)	Group 3 (post)	Baseline
PCV (%)	44.33 ± 1.53	38.00 ± 2.65	38.67 ± 1.53	39.00 ± 1.41	38.00 ± 2.65	35.33 ± 1.53	38-49
TWBC (10^3^/*μ*L)	5.87 ± 1.81	7.87 ± 3.35	4.27 ± 1.07	4.40 ± 1.70	3.40 ± 0.46	5.07 ± 1.12	3.1-11.8
MCHC (g/dL)	20.37 ± 0.86	25.33 ± 2.68	22.87 ± 2.17	24.85 ± 0.49	23.97 ± 2.46	19.93 ± 0.57	31.16-34.63
MCH (pg)	14.43 ± 0.76	16.07 ± 3.00	16.33 ± 0.42	16.45 ± 2.76	15.93 ± 1.40	14.03 ± 0.68	13.89-20.88
MCV (%)	71.40 ± 0.92	71.70 ± 2.95	71.03 ± 0.93	72.40 ± 2.69	71.03 ± 3.14	67.77 ± 1.56	44.0-65.0
PLT	552.67 ± 63.95	516.33 ± 46.54	574.33 ± 82.31	542.50 ± 14.85	581.67 ± 37.54	549.67 ± 47.04	480.0-725.0
Hb (g/dL)	14.90 ± 0.26	12.77 ± 1.07	13.27 ± 0.50	13.30 ± 0.57	12.97 ± 0.95	11.87 ± 0.70	13.77-16.00
Lymphocytes	73.67 ± 3.21	59.67 ± 1.53	73.00 ± 4.58	69.00 ± 7.07	68.33 ± 3.51	60.00 ± 6.00	56.1-78.0
Neutrophil	22.33 ± 3.21	39.00 ± 1.73	25.33 ± 5.03	30.00 ± 5.66	28.33 ± 4.04	29.33 ± 4.51	22.0-44.9
Monocytes	3.00 ± 1.00	1.33 ± 1.15	1.33 ± 1.15	1.00 ± 1.41	2.33 ± 0.58	2.00 ± 1.00	0.00-0.14
Eosinophil	1 ± 1	0	0.33 ± 0.58	0	0.67 ± 0.58	0.67 ± 1.15	0.00-0.11
Basophil	0	0	0	0	0	0	0.00-0.05

PCV: packed cell volume; TWBC: total white blood cell count; MCHC: mean corpuscular haemoglobin concentration; MCH: mean corpuscular haemoglobin; PLT: platelet; Hb: haemoglobin (values presented as the mean ± SD, *n* = 3).

## Data Availability

Data are available on demand.
